# A microprocessor controlled scanning polarograph for solution labile compounds

**DOI:** 10.1155/S1463924680000247

**Published:** 1980

**Authors:** R. E. Cooley, C. E. Stevenson, E. C. Rickard

**Affiliations:** The Lilly Research Laboratories Eli Lilly and Company P.O. Box 618 Indianapolis Indiana 46206 USA


### A microprocessor controlled scanning polarograph for solution labile compounds

**R. E. Cooley, C. E. Stevenson and E. C. Rickard**
*The Lilly Research Laboratories, Eli Lilly and Company, P.O. Box 618, Indianapolis, Indiana 46206, USA.*

### Introduction

The progressively greater number of analytical tests required on a sample and the larger number of samples required for significance of the data demand higher productivity to minimise increases in labour and equipment costs. At the same time, the precision and accuracy of the data must be maintained at the highest possible levels to assure validity of the results. Within the author's laboratories, automation of equipment has been used to meet these objectives of increased efficiency and high quality [1, 2, 3]. Polarography, one of the few routine analytical techniques not previously automated in the author's laboratory, is a labour and equipment intensive technique suitable for automation. This paper describes the development of an automated polarographic unit and compares its performance with non-automated procedures.

Two considerations are important in the polarographic assay procedures used. First, the cephalosporin antiobiotics, which comprise the largest quantity of polarographic samples tested [6], undergo decomposition in solution. Thus, an accurate determination of their purity is possible only when the sample is dissolved just prior to analysis [4, 5, 6]. Second, the solution concentration is directly related to the polarographic diffusion limited current which has been baseline corrected for residual current contributions. Two methods of corrections for residual currents are available: (1) extrapolation of the residual current baseline of the polarogram of the sample being analysed, and (2) measurement of the residual current on a blank solution. The former method corrects for residual currents due to impurities in the sample or other matrix effects but it is more complex than the latter technique where all currents are measured at a constant potential.

Several papers have been published describing automated polarographic systems. The largest number of these systems utilise available Auto-Analyzer (Technicon Instruments Corp, Tarrytown, New York) components. A three-part paper by Lund and Opheim [7, 8, 9] is a good example of such flow-through type systems. The dissolved sample is aspirated from the sample cup and pumped through the flow cell.

A less complex system described by Flann [10] utilizes a modified Technicon Sampler II. A redesigned sampler arm places the deaeration probes and the electrodes into the proper cups. Both of these systems utilize previously dissolved samples (i.e. aged solutions) and measure currents at a constant potential. A commercial unit (Automatic Cell Sequencer, model 316, E.G. & G. Princeton Applied Research, Princeton, New Jersey) works on a principle similar to Flann's system except that the entire polarogram is recorded.

A system which automates the dissolution, deaeration and scanning of the sample has been reported by Cullen et al [11]. The system has been used successfully to analyse pharmaceuticals in their finished form. The dissolution of the sample is accomplished with a Technicon Solidprep sampler. The Solidprep transfers the dry sample from the sample cup to the dissolution chamber. However, problems with this transfer operation have been documented [12, 13]. If the transfer of the sample is not complete, the accuracy and precision of the assay will be reduced.

The problems associated with the transfer of the dry sample from the sample cup to the dissolution chamber have been overcome by the authors' group by the development of a sampler unit which dissolves the samples in the removable glass weighing vessels. The design of the sampler also makes it possible to dissolve the sample just prior to analysis to minimise decomposition. The sampler unit has been interfaced with a 3-electrode polarograph (model 174A, E.G. & G. Princeton Applied Research, Princeton, New Jersey) for analysis of samples.

### Experimental

The automated system was controlled by interfacing the sampler and polarograph to an 8080 based microprocessor (model MMD-1, E. & L. Electronics, Derby, Conn., 06418). The functional block diagram of the system is shown in Figure 1. It automatically adds any combination of volumes of two possible solvents, mixes the sample, transfers it to the polarographic cell, deaerates and runs the polarographic scan. These operations can be carried out on a maximum of twenty-five samples before the tray must be reloaded.

To operate the polarograph under the control of the microprocessor, several front panel switches of the polarograph were disconnected and replaced with sub-miniature relays which have gold plated contacts (PZSM-D10058, ITT Components Group, North Andover, Mass., 01845) to minimise resistance. These relays control the "standby" and "scan" functions, make the necessary cell connections and control the recorder chart drive.

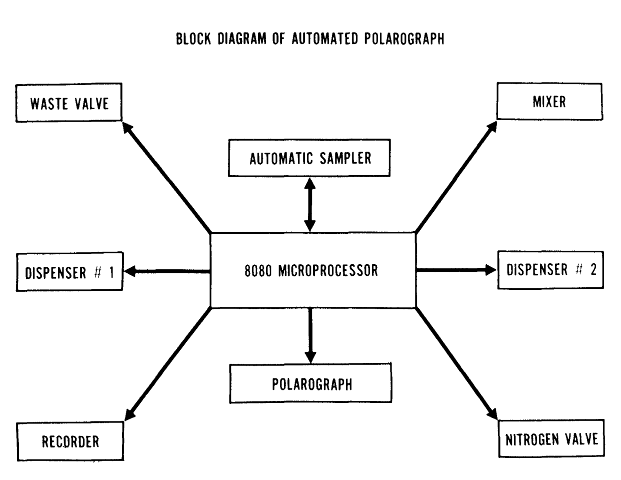

*Figure 1. Block diagram of automated system showing operations controlled by the microprocessor. Double arrow heads signify bidirectional data transfer.*

The sample tray and the sampler head are under the control of the microprocessor. Position sensing is performed by a system of photo-interruptors (G. E. H13B2, General Electric Co., Syracuse, New York 13201) and slotted disks. The positioning system of the turntable is shown in Figure 2. Disk A is attached directly to the motor shaft (model T, 60 rpm, Hurst Mfg. Corp., Princeton, Ind. 47670), and is used to sense the revolutions of the motor. Disk B and the turntable are attached to the motor through a 100 to 1 speed reducer (WX64B4-1, Winfred M. Berg, Inc., East Rockaway, New York). Disk B thus rotates at the same speed as the turntable. The turntable is positioned such that the slit in disk B passes through the photo-interruptor when position one on the turntable is below the dispenser head. The microprocessor checks for simultaneously low outputs from the photo-interruptors. This occurs when the slits in the two disks pass through the photo-interruptors concurrently. The motor is then immediately stopped so that the turntable is correctly positioned for the first sample.

Since the tray has twenty-five sample positions and the motor speed is reduced by a factor of 100, the motor must turn four revolutions between sample positions. The microprocessor will, therefore, see four interruptions from slit A for each sample position change on the turntable. This enables the microprocessor to determine the position of the turntable without the use of BCD position encoders. (The actual sample position will be equal to the number of interrupts observed divided by four). When samples are not assayed in numerical order, it also enables the microprocessor to determine whether clockwise or counterclockwise movement is the shortest distance to the next sample. Since the slit has a finite width, the turntable must always approach the sample position from the same side (clockwise rotation was selected). This is accomplished when moving counterclockwise by passing the sample position one revolution of disk A and then moving clockwise until the slit is seen again.

A similar position encoding system is used to enable the microprocessor to determine the vertical position of the sampler head (Figure 3). To initialise this positioning system at the beginning of each run, the microprocessor drives the sampler head upward while checking for a high output from photo-interruptor C. This state occurs when the wedge, attached to the sampler head, blocks the light beam from the photo-interruptor. The microprocessor then looks for the slit in disk D. When both conditions are satisfied, the motor (model T, 30 rpm, Hurst Mfg. Corp., Princeton, Ind. 47670) is stopped and the vertical position of the sampler head is set to zero in the microprocessor memory. Moving the sampler head to a new position requires calling the appropriate subroutine with an argument between 0 and 45 decimal in the proper register. This number corresponds to the number of interrupts the microprocessor would see from photo-interruptor D when moving the sampler head from the top position to the bottom position. The current position is stored so that the microprocessor can determine whether it must move up or down to reach the next position. Limit switches are mounted at the top and bottom of the sampler head drive to prevent over driving the head, should the microprocessor control ever fail. A limit switch is also mounted such that the turntable must have the sample tube positioned directly under the sampler head to activate the drive. This precaution prevents driving the head into the turntable in the event of turntable positioning control failure.

The transfer of the sample solution from the vessel is accomplished by driving the sampler head into the vessel, analogous to the plunger of a syringe. This procedure forces the sample out of the vessel through a hole machined in the centre of the Teflon sampler head. A groove machined in the end of the sampler head holds a replaceable silicone rubber O-ring that maintains the seal between the head and the vessel wall. To ensure reproducible sample sizes, the sample vessels were made from precision bore glass tubing.

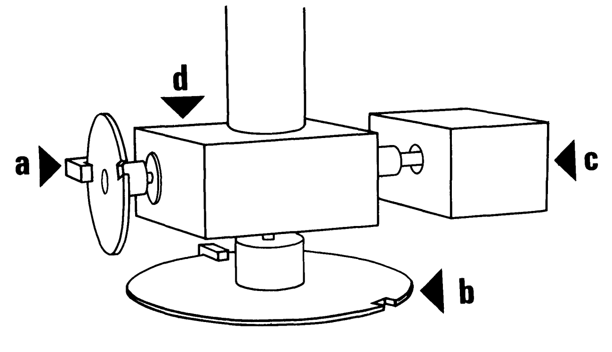

*Figure 2. Position sensing and drive mechanism of sample turntable.*
*a) sample position sensor, b) "home" position sensor, c) drive motor, d) speed reducer.*

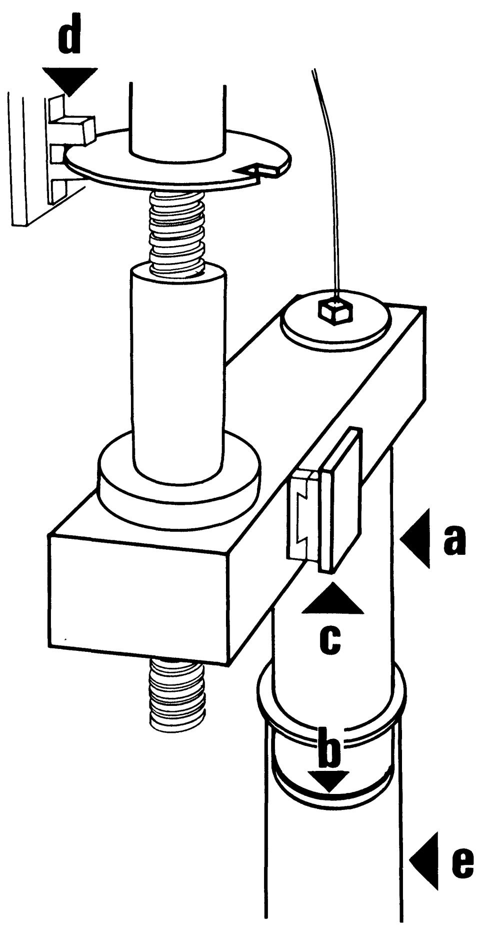

*Figure 3. Position sensing and drive mechanism of sampler head.*
*a) sampler head, b) removable O-ring, c) top position sensor, d) vertical position sensor, e) removable sample vial.*

The polarographic cell was constructed with a medium porosity frit in the bottom of the cell. The frit allows nitrogen to flow in such a manner as to obtain maximum deaeration. A flow of nitrogen is maintained over the surface of the sample through a connection on the side of the cell and flow direction is selected by the microprocessor. A hole in the centre of the frit is connected to a vacuum aspirator which drains the cell through a microprocessor controlled solenoid valve. The connection on the lower side of the cell is for entrance of the sample. Fine porosity frits were placed in the sides of the cell and connected to the auxiliary and reference electrode compartments.

### Procedure

The operation of the sampler may best be described by stepping through the analysis of a typical sample. The sample is weighed into the removable glass sample vessel on the balance and a magnetic stirring bar is added. The vessel is then placed on the sampler tray. The operating parameters; number of samples, minutes per scan, number of scans per sample and the volumes to be dispensed from each of two solvent reservoirs, are entered on the switch panel. The first sample vessel is positioned under the dispenser head and the selected amount of solvent added by the Hamilton syringe dispensers (Precision Liquid Dispenser, Hamilton Co., Reno, Nev. 89510). The sample vessel is then moved to the mixing station and stirred. If additional solvent is to be added, the vessel is returned to the dispenser station and the dispensing and mixing operations are repeated. Next, the sample is displaced to the cell where it is deaerated and scanned the selected number of times. Completion of the current sample initiates the sequence for the next sample, so that the length of time in solution is the same for all samples.

Possible sources of error induced by the use of this sampler were evaluated. Two obvious sources of possible error were (1) poor precision of the dispensers and (2) contamination of a sample by carryover of the previous sample, since the sampler head is not rinsed between samples.

Dispenser precision was evaluated by dispensing a constant volume of deionized water into nine weighed sample vials. The dispenser was set to deliver about 32 ml of deionised water by four injections of approximately 8 ml each. The accuracy of this volume was not checked since only its reproducibility is critical. After dispensing was complete, the vials were removed and reweighed.

The carryover was checked by alternately placing vials containing powdered methylene blue between empty vials. The dispenser was set to deliver approximately 32 ml of deionised water to each vial. The methylene blue was dissolved and displaced to the sample cell. The cell was drained and the same procedure was performed on the blank vial. This procedure was repeated on four pairs of vials. Samples were withdrawn from the vials containing methylene blue, diluted 1 ml to 250 ml with deionised water and the absorbance measured at 630 nm versus deionised water. The absorbance of the solution in the blank vial was measured after the sampler head had come in contact with it without prior rinsing.

The precision of the total system, when interfaced with the polarograph, was checked by assaying twenty-four replicate antibiotic samples. Approximately 14 mg of the powdered cephalosporin was weighed into each of twenty-four sample vials and placed on the sampler tray. The automatic system was then started. Twenty milliliters of water was added to dissolve the sample, followed by 30 ml of a pH 2.3 McIllvaine buffer. The sample solution was

DISPENSER PRECISION

| Weight Of Solution Delivered (gm) |
|---|
| 31.3016 |
| 31.3146 |
| 31.3047 |
| 31.3072 |
| 31.3053 |
| 31.3048 |
| 31.3094 |
| 31.3041 |
| 31.3001 |

MEAN = 31.3058 RSD = 0.014 %

*Table 1. Dispenser precision.*

AUTOMATED POLAROGRAPH SYSTEM PRECISION

Ratio Of Sample Weight To Wave Height

| | | |
|---|---|---|
| 0.8795 | 0.8839 | 0.8843 |
| 0.8741 | 0.8855 | 0.8736 |
| 0.8865 | 0.8847 | 0.8699 |
| 0.8716 | 0.8755 | 0.8690 |
| 0.8827 | 0.8821 | 0.8735 |
| 0.8798 | 0.8755 | 0.8791 |
| 0.8803 | 0.8730 | 0.8760 |
| 0.8760 | 0.8803 | 0.8809 |

Mean = 0.8782 RSD = 0.58 %

*Table 2. Precision of entire system when used to analyse a cephalosporin antibiotic in the differential pulse polarographic mode.*

mixed and two portions were displaced to the cell for rinsing. A third portion was displaced and retained in the cell where it was deaerated for five minutes with nitrogen prior to assay. Each sample was scanned, in the differential pulse polarographic mode, three times.

### Results and discussion

The precision of the liquid dispensers was found to be quite adequate for this application (Table 1). The mean weight delivered was 31.31 g with a relative standard deviation of less than 0.014%.

The carryover in the blank vial was found to be 0.05% of the previous sample's absorbance. Based on this data, rinsing of the sampler head was felt to be unnecessary. The carry-over found in the cell, after the initial transfer of the next sample, was approximately four times higher due to incomplete draining of the cell and transfer tubing. To minimise this error, the cell is rinsed twice with the sample prior to filling for analysis.

The overall system precision was calculated from the average of the three replicate scans after correcting for differences in sample weight (Table 2). The relative standard deviation in the differential pulse polarographic mode was 0.58%. When only one scan per sample was used to calculate precision, the relative standard deviation was 0.62%. In the differential pulse polarographic mode, an analysis of a sample versus a standard will give a precision of 0.85% (for three scans) since the total variance will contain contributions from both the sample and the standard. Polarography in the sampled DC (Tast) mode gave comparable precision with linearity over a wider concentration range.

### Conclusions

The reproducibility of the automated polarograph has been shown to be better than the 1.6% expected for a manual assay [4]. Moreover, by using the automated polarograph in the 'silent hours', one can assay 40 samples per day with three replicate scans per sample. When only one scan per sample is required, the time can be reduced from approximately 30 minutes per sample to 20 minutes per sample. When operating the polarograph in a manual mode, only about 10-12 samples can be completed in one work shift. Thus, the automated system has more than tripled the throughput and has resulted in increased precision.

Although the sampler was used in this application with a polarograph, it was designed to be used with essentially any instrument that utilises a dissolved sample for analysis. Each operation of the sampler (eg dispensing, sampling, deaeration, scanning, etc) is written into software subroutines so that reprogramming of the controller for other applications requires only a rearrangement of subroutine calls and redefinition of the functions of the front panel switches. Planned applications of the sampler in the authors' laboratories include the analysis of antibiotics by liquid chromatography and the preparation of UV-visible spectra of standard materials.

ACKNOWLEDGMENTS

The authors wish to express their appreciation to Mr. H. Pierson for the machining of the sampler parts and his valuable advice during its construction. We would also like to thank Mr. R. Miller for his work in the construction of the polarographic cell and sample vessels. Suggestions by Mr M. Skibic were most helpful. The technical assistance of Messrs P. Farb and E. Pagel is also acknowledged.

REFERENCES

[1] Cooley, R. E., *Analytical Chemistry*, 1978, **50**, 1231-1232.

[2] Cooley, R. E., "A Microprocessor Controlled Sample For The Automatic Collection and Filtration of Dissolution Samples," presented at the Federation of Analytical Chemistry and Applied Spectroscopy Societies meeting, Sept., 1979, Philadelphia, Pennsylvania.

[3] Stevenson, C. E., Lorenz, L. J. and Coffey, H. F., "A Liquid Sampling Device for Liquid Chromatography", presented at the 1975 Pittsburgh Conference On Analytical Chemistry And Applied Spectroscopy, March, 1975, Cleveland, Ohio.

[4] Rickard, E. C. and Cook, G. C., *Journal of Pharmaceutical Science* 1977, **66**, 379

[5] Kaiser, G. V., Gorman, M. and Allan Webber, J., *Journal Of Infectious Diseases*, 1978, **127**, 510

[6] Bornstein, M., Thomas, P. N., Coleman, D. L. and Boylan, J. C., *American Journal of Hospital Pharmacy*, 1974, **31**, 296

[7] Lund, W. and Opheim, L., *Analytica Chimica Acta*, 1975, 79, 35-45

[8] Lund, W. and Opheim, L., *Analytica Chimica Acta*, 1975, 79, 245-254

[9] Lund, W. and Opheim, L., *Analytica Chimica Acta*, 1977, 88, 241-259

[10] Flann, B., *Chemical Instrumentation*, 1979, 7, 4, 241-259

[11] Cullen, L. F., Brindle, M. P. and Papariello, J.G., *Advances in Automated Analysis*, **9**, Technicon International Congress 1972, Mediad, Inc., Tarrytown, New York 10591.

[12] Ahuja, S., Spitzer, C. and Brofazi, F. R., *Automation In Analytical Chemistry*, 1967, **1**, Technicon Symposia, Mediad, Inc., White Plains, New York, p. 439

[13] Ahuja, S., Spitzer, C. and Brofazi, F. R., *Automation In Analytical Chemistry*, 1967, **1**, Technicon International Symposia, Mediad, Inc., White Plains, New York, p. 467

**Automatic Chemical Analysis Summer School**

An intensive residential course in all aspects of automatic chemical analysis will be held in the Chemistry Department of the University College of Wales, Swansea from the 6th to the 11th July 1980. The course will be of interest to technologists already using automated methods; those wishing to evaluate the potential of automation; postgraduate students; and managers who need to understand the role of automation. The programme will include lectures and practical sessions. Application for further information should be made to: Dr. P.B. Stockwell, Laboratory of the Government Chemist, Cornwall House, Stamford Street, LondonSE1 9NQ

